# Supplementary material for: CHSI costing study–Challenges and solutions for cost data collection in private hospitals in India
Source: PLoS One. 2022 Dec 12;17(12):e0276399. doi: 10.1371/journal.pone.0276399 (PMC9744278; doi:10.1371/journal.pone.0276399)
Supplement: S1 Table — (DOCX) [file pone.0276399.s001.docx]

**Article Title: CHSI costing study – Challenges and solutions for cost data collection in private hospitals in India**

**Authors:** Maninder Pal Singh^1,4^, Riya Popli^1^, Sehr Brar^1^, Kavitha Rajsekar^3^, Oshima Sachin^3^, Jyotsna Naik^3^, Sanjay Kumar^5^, Setu Sinha^5^, Varsha Singh^5^, Prakash Patel^6^, Ramesh Verma^7^, Avijit Hazra^8^, Raghunath Misra^8^, Divya Mehrotra^9^, Sashi Bhusan Biswal^10^, Ankita Panigrahy^10^, Kusum Lata Gaur^11^, Jai Prakash Pankaj^11^, Dharmesh Kumar Sharma^11^, Kondeti Madhavi^12^, Pulaganti Madhusudana^12^, K. Narayanasamy^13^, A. Chitra^13^, Gajanan D Velhal^14^, Amit S Bhondve^14^, Rakesh Bahl^15^, Sharminder Kaur^15^, Shankar Prinja^1,2*^

**Supporting Table S1: Costing of Health Services (CHSI) Study Sampling**

A multistage stratified sampling method was used. The states were selected to represent the heterogeneity based on geography, health indicators, net state domestic product (NSDP) and health workforce density. The state specific factors are i.e. population, geography, Sustainable Development Goals India Index, Human Development Index, per capita NSDP and health workforce density (per 10,000 population) are shown in the table below for the sampled states.

Table 1: Costing of Health Services (CHSI) Sampling Framework

| **State** | **Population (Census 2011)** | **Geography** | **SDG India Index 2018***  **(0-100)** | **Human Development Index**  **(HDI)** | **Gross State Domestic Product#**  **(GSDP)** | **Health workforce Density** |
| --- | --- | --- | --- | --- | --- | --- |
| **Jammu & Kashmir** | 12,541,302 | North | 53 | Medium | Low | Medium |
| **New Delhi** | 18,345,784 | North | 62 | High | High | High |
| **Rajasthan** | 68,548,437 | North | 59 | Low | Low | Low |
| **Uttar Pradesh** | 199,812,341 | North | 48 | Low | Low | Low |
| **Gujarat** | 60,439,692 | West | 64 | Medium | Medium | Medium |
| **Maharashtra** | 112,374,333 | West | 64 | Medium | High | High |
| **Andhra Pradesh** | 84,580,777 | South | 64 | Medium | Medium | Medium |
| **Tamil Nadu** | 72,147,030 | South | 66 | High | High | High |
| **Bihar** | 104,099,452 | East | 42 | Low | Low | Low |
| **West Bengal** | 91,276,115 | East | 56 | Low | Low | High |
| **Odisha** | 41,974,218 | East | 51 | Low | Low | Low |
| **Meghalaya** | 2,966,889 | North-East | 52 | Medium | Low | Low |
| *SDG: Sustainable Development Goals; Source: NITI Aayog 2018, ^#^Reserve Bank of India | | | | | | |
| *Source: Prinja S, Singh MP, Guinness L, Rajsekar K, Bhargava B. Establishing reference costs for the health benefit packages under universal health coverage in India: cost of health services in India (CHSI) protocol. BMJ Open. 2020;10(7):e035170.* | | | | | | |

Within each state, a tertiary level medical institution was chosen. At the secondary care level, costing was done at three district hospitals in each state. The districts were randomly selected from each of the three tertiles of the district composite development score ranking. This index was based on an aggregation of socioeconomic, demographic and health service usage indicators.’
