# Supplementary material for: CHSI costing study–Challenges and solutions for cost data collection in private hospitals in India
Source: PLoS One. 2022 Dec 12;17(12):e0276399. doi: 10.1371/journal.pone.0276399 (PMC9744278; doi:10.1371/journal.pone.0276399)
Supplement: S2 Table — (DOCX) [file pone.0276399.s002.docx]

**Article Title: CHSI costing study – Challenges and solutions for cost data collection in private hospitals in India**

**Authors:** Maninder Pal Singh^1,4^, Riya Popli^1^, Sehr Brar^1^, Kavitha Rajsekar^3^, Oshima Sachin^3^, Jyotsna Naik^3^, Sanjay Kumar^5^, Setu Sinha^5^, Varsha Singh^5^, Prakash Patel^6^, Ramesh Verma^7^, Avijit Hazra^8^, Raghunath Misra^8^, Divya Mehrotra^9^, Sashi Bhusan Biswal^10^, Ankita Panigrahy^10^, Kusum Lata Gaur^11^, Jai Prakash Pankaj^11^, Dharmesh Kumar Sharma^11^, Kondeti Madhavi^12^, Pulaganti Madhusudana^12^, K. Narayanasamy^13^, A. Chitra^13^, Gajanan D Velhal^14^, Amit S Bhondve^14^, Rakesh Bahl^15^, Sharminder Kaur^15^, Shankar Prinja^1,2*^

**Supporting Table S2: Costing of Health Services (CHSI) Time Allocation Tool**

Staff Member Code (Enter Code as entered in Section 1): ............................................

| **Service code no** | **Activity name** | **Type of activity** | | **Fixed schedule activity** | | | **Routine activity** | | |
| --- | --- | --- | --- | --- | --- | --- | --- | --- | --- |
|  |  | **Fixed schedule** | **Routine** | **Frequency (once in a week/once in month/twice a week etc.) *** | **Hours per day of activity** | **Days for which the activity was done during the reference** **year** | **Time per person (in minutes) (a)** | **Number of beneficiaries on a routine day (b)** | **If not (a) and (b) then how much time to do the activity** |
|  | Outpatient (OP) department |  |  |  |  |  |  |  |  |
|  | Inpatient (IP) department |  |  |  |  |  |  |  |  |
|  | Intensive care unit (ICU) |  |  |  |  |  |  |  |  |
|  | Operation Theatre (OT) |  |  |  |  |  |  |  |  |
|  | General Administration |  |  |  |  |  |  |  |  |
|  | Teaching/Training |  |  |  |  |  |  |  |  |
|  | Workshop/Conference |  |  |  |  |  |  |  |  |
|  | Outreach |  |  |  |  |  |  |  |  |
|  | Meetings |  |  |  |  |  |  |  |  |
|  | Research |  |  |  |  |  |  |  |  |
|  | Others (Specify) |  |  |  |  |  |  |  |  |
| - **Codes: ‘1’ for once a year participation, ‘2’ for twice a year, 3 for thrice a year participation, 4 for quarterly participation, 5 for once every two** - **months, 6 for monthly participation, 7 for fortnightly participation, 8 for weekly participation, 9 for twice a week participation, 10 for thrice** - **a week participation.** | | | | | | | | | |
