# Supplementary material for: CHSI costing study–Challenges and solutions for cost data collection in private hospitals in India
Source: PLoS One. 2022 Dec 12;17(12):e0276399. doi: 10.1371/journal.pone.0276399 (PMC9744278; doi:10.1371/journal.pone.0276399)
Supplement: S3 Table — (DOCX) [file pone.0276399.s003.docx]

**Article Title: CHSI costing study – Challenges and solutions for cost data collection in private hospitals in India**

**Authors:** Maninder Pal Singh^1,4^, Riya Popli^1^, Sehr Brar^1^, Kavitha Rajsekar^3^, Oshima Sachin^3^, Jyotsna Naik^3^, Sanjay Kumar^5^, Setu Sinha^5^, Varsha Singh^5^, Prakash Patel^6^, Ramesh Verma^7^, Avijit Hazra^8^, Raghunath Misra^8^, Divya Mehrotra^9^, Sashi Bhusan Biswal^10^, Ankita Panigrahy^10^, Kusum Lata Gaur^11^, Jai Prakash Pankaj^11^, Dharmesh Kumar Sharma^11^, Kondeti Madhavi^12^, Pulaganti Madhusudana^12^, K. Narayanasamy^13^, A. Chitra^13^, Gajanan D Velhal^14^, Amit S Bhondve^14^, Rakesh Bahl^15^, Sharminder Kaur^15^, Shankar Prinja^1,2*^

**Supporting Table S3: Private sector process evaluation survey for Costing of Health Services in India (CHSI) study**

**COSTING OF HEALTH SERVICES IN INDIA (CHSI)**

**PROCESS DOCUMENTATION SURVEY**

**PRIVATE SECTOR**

# Background and purpose of the survey

The DHR multi-site costing study is the first national level survey of health system costs to be carried out in India. As part of this endeavour, it is critical to ensure lessons are learned that will feed into methodological improvement, the robustness of the cost data as well as help explain variations in unit costs across different facilities. Retrospective cost data collection requires decisions that are context specific and relies on making sensible decisions where full data is not available. This results in some methodological variation across sites. To enable comparability of cost data these variations need to be described. This survey is being carried out to document the process of data collection and capture the methodological choices made as well as document challenges faced and how these have been addressed. The survey results will be used to ensure comparability of costs across sites and to develop recommendations for future research in this area.

# Structure of the tool

**Section 1: MRU Details**- This section requires you to fill all MRU, project in-charge and department details.

**Section 2: Input Processes and Challenges**- This section focusses on mainly two inputs of the project, namely institutional permissions and recruitment & training of staff. Please answer all objective and subjective questions. Feel free to add any additional comments on variations in different departments.

**Section 3: Data wise processes and Challenges**- This section requires detailed information on the data collection process and challenges faced, according to each type of data collected.

- **Part 3A:** Contains multiple choice questions related to collection of different types of data. Multiple options may be selected in each question. For selecting the appropriate options highlight in yellow.
- **Part 3B**: In this part there are 9 columns to be filled for each type of data:
- **Time taken to complete data collection**: Please mention both, the time spent on negotiation and waiting for data and the actual time of extraction of that particular data.
- **Data Source – Level of aggregation**: Mention the level at which this data was available, such as for the whole institution, for the department or for the specific cost center (ICU/OPD/OT/IPD)
- **Data Source – Type of data source:** Mention the form of the data – Electronic/ physical registers/conversation with personnel etc.
- **Person contacted:** Mention the person contacted for that particular data and also the person who found to be most suitable for obtaining the data (incase multiple people were contacted). Name of the person is not required; the designation would suffice.
- **Name of personnel collecting data:** Mention the designation and name of person who collected the data – Investigator/Co-Principal Investigator/Admin staff etc.
- **Challenges faced:** Please mention in detail any challenges that were faced during the collection or retrieval of the data. Provide details of the same.
- **Innovative ideas used to tackle the problem:** Please mention any ideas which were used to tackle the challenge and obtain the data.
- **How would you rate the difficulty of data collection on a Scale of 1 to 10:** Rate the level of challenge on a scale of 1 to 10 where 1 implies no difficulty and 10 implies not possible to collect data.
- **Part 3C**: Details of data collected for OT procedures. Similar to Section 3B, information is to be entered related to time taken to collect data, person contacted, person collecting data, challenges faced, innovative ideas and rating of difficulty.

**Section 4: Training and supervision for cost data collection:** This section focusses on the training and supervisory support provided by PGIMER team. Please provide your inputs on the subjective questions asked. Kindly provide your feedback freely to improve the trainings and support further.

**Section 5: Suggestions-** This section focusses on your inputs for the improvement of data collection from your experience as one of the data collection sites.

# General instructions

- This tool is to be filled in this document in **MS Word** itself. Please do not send any hard copy of the filled format to PGIMER team, you are required to complete the tool in the soft copy and **share it by email**.
- Please answer all questions as fully as possible.
- ONE tool is to be filled for each institution including all allotted departments.
- For objective (multiple choice questions) highlight the all appropriate option to selected in YELLOW.
- If there are additional challenges that you would like to share please do so at the end of the survey.

# **SECTION 1: MRU DETAILS**

**NAME OF MRU:**

**STATE**:

**YEAR OF ESTABLISHMENT OF MRU:**

**NAME OF TERTIARY HOSPITAL**:

**BED CAPACITY OF HOSPITAL**:

**NAME, DESIGNATION & CONTACT OF COODINATOR**:


**DEPARTMENTS ALLOTTED:**

| **Department Allotted** | **Date of initiation of data collection** | **Date of completion of data collection** | **Date of completion of Data entry** | **Date of submission of queries** | **Date Of final data sharing** |
| --- | --- | --- | --- | --- | --- |
|  |  |  |  |  |  |
|  |  |  |  |  |  |
|  |  |  |  |  |  |
|  |  |  |  |  |  |
|  |  |  |  |  |  |
|  |  |  |  |  |  |
|  |  |  |  |  |  |
|  |  |  |  |  |  |
|  |  |  |  |  |  |
|  |  |  |  |  |  |

**DEPARTMENT ANNUAL LOAD DETAILS**

| **Name of Department** | **OPD** | | **IPD** | | **ICU** | | **Surgeries in OT** | |
| --- | --- | --- | --- | --- | --- | --- | --- | --- |
|  | **Total number** | **Source** | **Total number** | **Source** | **Total number** | **Source** | **Total number** | **Source** |
| **1** |  |  |  |  |  |  |  |  |
| **2** |  |  |  |  |  |  |  |  |
| **3** |  |  |  |  |  |  |  |  |
| **4** |  |  |  |  |  |  |  |  |
| **5** |  |  |  |  |  |  |  |  |
| **6** |  |  |  |  |  |  |  |  |
| **7** |  |  |  |  |  |  |  |  |
| **8** |  |  |  |  |  |  |  |  |
| **9** |  |  |  |  |  |  |  |  |
| **10** |  |  |  |  |  |  |  |  |

# **SECTION 2: INPUTS WISE PROCESSES & CHALLENGES**

**(Please HIGHLIGHT the appropriate options)**

**INSTITUTIONAL PERMISSIONS (Please respond for each set of institutional permissions that were obtained)**

1. Who was approached for obtaining institutional permissions?
   1. Dean/ Director/ Administrator of the institution
   2. Head of the Department
   3. Cost centre (OPD/IPD/ICU/OT) In-charge
   4. Any other, please specify:
2. Were any additional processes required to obtain permission?
   1. Institutional ethics approval
   2. Institutional Collaborative Research Committee approval
   3. Any other, please specify:
3. What type of an interaction was made for permissions?
   1. Stakeholder meeting
   2. One on one meeting with the in-charge of the facility
   3. One on one meeting with individual cost centre in-charge
   4. Any other, please specify:
4. **How much time did permissions take before the work could be started (in days)?**
5. **Were there any problems in obtaining permission? If so, how were they handled?**
6. Were you ultimately denied permission in any department? If yes, how many?

## **RECRUITMENT AND TRAINING OF STAFF**

1. How many eligible candidates were interviewed for the post of Field Investigator?
2. Where there any problems in recruiting staff? Please describe these problems and how you resolved them.

**PROJECT STAFF DETAILS:** Please list all the staff involved in the cost data collection and analysis at your MRU whether they are/were full time or part-time

| **Name** | **Designation** | **Date of appointment** | **Qualification & Experience** | **Type of duties entrusted** | **Trained for data collection – Yes/No** | **If trained, where?**  **PGIMER/Local training** | **If resigned, reason of resignation** |
| --- | --- | --- | --- | --- | --- | --- | --- |
|  |  |  |  |  |  |  |  |
|  |  |  |  |  |  |  |  |
|  |  |  |  |  |  |  |  |
|  |  |  |  |  |  |  |  |
|  |  |  |  |  |  |  |  |

# **SECTION 3: DATA TYPE WISE PROCESSES & CHALLENGES**

**Part 3A: PROCESS QUESTIONS (Highlight all applicable options)**

1. The information on salary was collected from:

A. Administration

B. Accounts branch

C. Person him/herself

D. Online data bases

E. Any other, please specify:

1. How many people were interviewed for time allocation on an average for each type of staff, how many were with concerned person and how many with proxy (A staff member who knew the schedule of the concerned person).

Name your departments and mention details for each of them.

|  |  | **Department 1** | **Department 2** | **Department 3** | **Department 4** |
| --- | --- | --- | --- | --- | --- |
| **1** | **CONSULTANTS** |  |  |  |  |
|  | Person concerned |  |  |  |  |
|  | Proxy |  |  |  |  |
| **2** | **SENIOR RESIDENTS** |  |  |  |  |
|  | Person concerned |  |  |  |  |
|  | Proxy |  |  |  |  |
| **3** | **JUNIOR RESIDENTS** |  |  |  |  |
|  | Person concerned |  |  |  |  |
|  | Proxy |  |  |  |  |
| **4** | **NURSING STAFF** |  |  |  |  |
|  | Person concerned |  |  |  |  |
|  | Proxy |  |  |  |  |
| **5** | **SUPPORT STAFF** |  |  |  |  |
|  | Person concerned |  |  |  |  |
|  | Proxy |  |  |  |  |
| **6** | **ANY OTHER STAFF** |  |  |  |  |
|  | Person concerned |  |  |  |  |
|  | Proxy |  |  |  |  |

1. Were time allocation tables checked for tally of total number of working hours and days?

A. Yes, and they matched

B. Yes, and they didn’t match – how did you resolve this?

C. No

1. The area of the facility was determined from (please highlight ALL applicable options in yellow):

A. Architecture/administrative department

B. Blue print of building

C. Physical measurement by investigator

D. Using measurement aids

E. Any other, please specify:

1. Was the process of measuring space similar across all cost centres?
   1. Yes
   2. No

If no, please mention all methods used and the reasons for using the different methods:

1. How was the rental price determined?

A. Market survey

B. Expert opinion

C. Estimation of housing rental in same area

D. Standard government charges

E. Any other, please specify:

If a market survey was done, how many key informants were interviewed? Specify the type of key informants.

1. Was data on consumables available at (please highlight ALL applicable options in yellow):
2. Patient level
3. Cost centre level (OT/ICU/OPD)
4. Departmental level (e.g. Ortho/OBG/ENT etc.)
5. Institution level (Hospital)
6. What was the source of prices of consumables? highlight ALL applicable options in yellow and mention the proportion of items for which price was determined from each source:

| **SOURCE** | **PROPORTION** |
| --- | --- |
| 1. Departmental store |  |
| 1. Central store/ Procurement office |  |
| 1. Chemist |  |
| 1. Online sources |  |
| 1. Any other, please specify: |  |

1. List of equipment and furniture items was obtained by;

|  | **EQUIPMENT** | **FURNITURE ITEMS** |
| --- | --- | --- |
| 1. Physical observation |  |  |
| 1. Records |  |  |
| 1. Both |  |  |

1. Was the functional status of each furniture item and equipment item cross checked through observation?

| **RESPONSE** | **EQUIPMENT** | **FURNITURE ITEMS** |
| --- | --- | --- |
| 1. Yes |  |  |
| 1. No |  |  |
| 1. Other methods used for cross-check? If so please specify? |  |  |

1. What was the source of prices of equipment and non-consumable items? highlight ALL applicable options in yellow and mention the proportion of items for which price was determined from each source:

| **SOURCE** | **EQUIPMENT** | **FURNITURE ITEMS** |
| --- | --- | --- |
|  | **PROPORTION** | **PROPORTION** |
| 1. Departmental store |  |  |
| 1. Central store/ Procurement office |  |  |
| 1. Online sources |  |  |
| 1. Any other, please specify: |  |  |

1. How was the information on average life of equipment and non-consumable items obtained? highlight ALL applicable options in yellow and mention proportions for each source.

| **SOURCE** | **EQUIPMENT** | **FURNITURE ITEMS** |
| --- | --- | --- |
|  | **PROPORTION** | **PROPORTION** |
| 1. Expert opinion |  |  |
| 1. Manual of item |  |  |
| 1. Online sources |  |  |
| 1. Review of literature |  |  |
| 1. Any other, please specify: |  |  |

1. How was the usage of equipments in different procedure determined?

| **RESPONSE** | **PROPORTION** |
| --- | --- |
| 1. A. By observation |  |
| B. Interview of concerned staff |  |
| 1. C. Records |  |
| 1. D. Any others, please specify: |  |

1. At what level was data on charges for the below mentioned overhead costs available?

| **Overhead** | **Cost head level (OT/ICU/OPD)** | **Departmental level (E.g. Ortho/OBG/EYE etc.)** | **Institution level (Hospital)** |
| --- | --- | --- | --- |
| Electricity |  |  |  |
| Building maintenance |  |  |  |
| Equipment Maintenance |  |  |  |
| Laundry |  |  |  |
| Dietetics |  |  |  |
| Biomedical waste |  |  |  |

1. What was the mode of operation of additional services of the hospital?

| **Service** | **Was the service outsourced or managed by hospital? (Outsourced/in-house)** | **If managed by hospital, was costing done for the department? (Yes/ No)** |
| --- | --- | --- |
| Laundry |  |  |
| Dietetics |  |  |
| Biomedical waste management |  |  |

1. How was biomedical waste management charged?

A. Per bed day

B. Yearly tender

C. Any other, please specify:

1. How many people were interviewed to collect the below mentioned data for OT procedures, department wise?

| **Type of data** | **Number of people interviewed** | | | |
| --- | --- | --- | --- | --- |
|  | **Name of department** | **Name of department** | **Name of department** | **Name of department** |
| **Average time of each procedure** |  |  |  |  |
| **Diagnostics used in each procedure** |  |  |  |  |
| **Average OPD visits (pre and post procedure)** |  |  |  |  |
| **List of drugs and consumables purchased by patient before procedure** |  |  |  |  |

1. How was data on Average length of stay in IPD collected?

A. Expert opinion

B. Patients records

1. If patient records were reviewed to determine average length of stay, in the given reference year, how many patient records were reviewed for data on average length of stay in IPD, department wise?

|  | **Number of patient records reviewed for average length of stay** |
| --- | --- |
| **Name of department 1** |  |
| **Name of department 2** |  |
| **Name of department 3** |  |
| **Name of department 4** |  |

1. How was data on Average length of stay in ICU collected?

A. Expert opinion

B. Patients records

1. If patient records were reviewed to determine average length of stay, in the given reference year, how many patient records were reviewed for data on average length of stay in ICU, department wise?

|  | **Number of patient records reviewed for average length of stay** |
| --- | --- |
| **Name of department 1** |  |
| **Name of department 2** |  |
| **Name of department 3** |  |
| **Name of department 4** |  |

1. Was any extra information collected for areas that were shared by multiple departments?

If yes which information?

•

•

## **Part 3B: DETAILS OF ALL TYPES OF DATA COLLECTED**

| **Type of Data** | **Time Taken to Complete data collection (in days)** | | **Data Source** | | **Person Contacted for Information (Mention all if multiple were contacted)** | **Best Person for Obtaining Information** | **Name of personnel Collecting the Data** | **Did you face any challenges?**  **(Yes/No)**  **Please describe.** | **Innovative ideas used to tackle the problem** | **How would you rate the difficulty to collect this data on a Scale of 1 to 10*** |
| --- | --- | --- | --- | --- | --- | --- | --- | --- | --- | --- |
|  | **Negotiating and waiting time (days):** | **Actual extraction time (days):** | **Level of Aggregation**  **(Institution/Department/ ICU/OPD/OT)** | **Type of source (Physical registers/electronic record database /Patient record sheets/personnel interview etc.)** |  |  |  |  |  |  |
| **HR Salary & Incentives** |  |  |  |  |  |  |  |  |  |  |
| **Leave data** |  |  |  |  |  |  |  |  |  |  |
| **Time allocation** |  |  |  |  |  |  |  |  |  |  |
| **Building area measurement** |  |  |  |  |  |  |  |  |  |  |
| **Determination of rental price** |  |  |  |  |  |  |  |  |  |  |
| **Consumables used** |  |  |  |  |  |  |  |  |  |  |
| **Prices of consumables** |  |  |  |  |  |  |  |  |  |  |
| **Furniture items used** |  |  |  |  |  |  |  |  |  |  |
| **Prices of non-consumable items** |  |  |  |  |  |  |  |  |  |  |
| **Information on average life of furniture items** |  |  |  |  |  |  |  |  |  |  |
| **Equipment used** |  |  |  |  |  |  |  |  |  |  |
| **Equipment procurement prices** |  |  |  |  |  |  |  |  |  |  |
| **Average life of equipment** |  |  |  |  |  |  |  |  |  |  |
| **Usage of equipment in different procedures** |  |  |  |  |  |  |  |  |  |  |
| **Electricity** |  |  |  |  |  |  |  |  |  |  |
| **Building maintenance** |  |  |  |  |  |  |  |  |  |  |
| **Equipment maintenance** |  |  |  |  |  |  |  |  |  |  |
| **Laundry** |  |  |  |  |  |  |  |  |  |  |
| **Dietetics** |  |  |  |  |  |  |  |  |  |  |
| **Biomedical waste management** |  |  |  |  |  |  |  |  |  |  |
| **Annual load data (OPD/IPD/ Surgeries)** |  |  |  |  |  |  |  |  |  |  |
| **Any other data collected, please specify:** |  |  |  |  |  |  |  |  |  |  |

## **Part 3C: DETAILS OF DATA COLLECTED FOR OT PROCEDURES**

| **Type of data** | **Time Taken to Complete data collection (in days)** | | **Person Contacted for Information (Mention all, if multiple were contacted)** | **Best Person for Obtaining Information** | **Designation of Personnel Collecting the Data** | **Did you face any challenges?**  **(Yes/No)**  **Please describe.** | **Innovative ideas used to tackle the problem** | **How would you rate the difficulty to collect this data on a Scale of 1 to 10*** |
| --- | --- | --- | --- | --- | --- | --- | --- | --- |
|  | **Negotiating and waiting time (days):** | **Negotiating and waiting time (days):** |  |  |  |  |  |  |
| **Average time of each procedure** |  |  |  |  |  |  |  |  |
| **Average length of stay in ICU and IPD** |  |  |  |  |  |  |  |  |
| **Diagnostics used in each procedure** |  |  |  |  |  |  |  |  |
| **Average OPD visits (pre and post procedure)** |  |  |  |  |  |  |  |  |
| **List of drugs and consumables purchased by patient before procedure** |  |  |  |  |  |  |  |  |

**Any other challenges/issues (Please describe any other challenges you had and how these were resolved):**

# **SECTION 4: TRAINING AND SUPERVISION FOR COST DATA COLLECTION**

| **TRAINING FOR COST DATA COLLECTION** | |
| --- | --- |
| **What did you feel were the best aspects of the training? Please explain why.** |  |
| **In which areas would you have liked more training? Please explain why.** |  |
| **Do you have any other comments about the training?** |  |
| **SUPERVISORY SUPPORT** | |
| **What did you feel were the best aspects of the supervisory support? Please explain why.** |  |
| **In which areas would you have liked more supervisory support? Please explain why.** |  |
| **Do you have any other comments about the supervisory support?** |  |

# **SECTION 5: SUGGESTIONS (Specific to Private Sector)**

What suggestions do you have to improve the ease of data collection? Your inputs maybe on:

|  | **SUGGESTIONS** |
| --- | --- |
| **Facility level issues** |  |
| **Departmental issues** |  |
| **Cost Centre level (OPD/OT/ICU/OPD)** |  |
| **Input Resource level (Like equipment, drugs etc.)** |  |
| **Operational Problems** |  |
| **Any other** |  |
