# Supplementary material for: CHSI costing study–Challenges and solutions for cost data collection in private hospitals in India
Source: PLoS One. 2022 Dec 12;17(12):e0276399. doi: 10.1371/journal.pone.0276399 (PMC9744278; doi:10.1371/journal.pone.0276399)
Supplement: Attachment — Submitted filename: Comments_Plos_One.docx [file pone.0276399.s005.docx]

It is an important and difficult study to dive in private sector. It is important to mention that it is a good effort. However, manuscript needs some additions for better clarity.

Please see if you can rework the title as “Challenges and solutions of inclusion of private hospitals in Costing of services” OR “Situational analysis for inclusion of private hospitals in Costing of Health Services in India”

**Introduction**

1. Include state of private hospitals in India, like number of hospitals. Categorization of hospitals (large and small) would be useful.

**Methods:**

1. Mention inclusion criteria of hospitals for the study.
2. Mention total number of private hospitals in each included state.
3. Mention refusal rate (overall and in each state), if not state the potential reasons of non-refusal.

**Discussion:**

Introduce limitation section in discussion section focusing;

1. Discuss about potential selection and observer bias in present study.
2. Whether results are generalizable or not?

If possible, a small review of legal challenges (if any) can be included to assess potential conflict if data sharing made mandatory by private hospitals.
